# Evaluation of an Ultraviolet C (UVC) Light-Emitting Device for Disinfection of High Touch Surfaces in Hospital Critical Areas

**DOI:** 10.3390/ijerph16193572

**Published:** 2019-09-24

**Authors:** Beatrice Casini, Benedetta Tuvo, Maria Luisa Cristina, Anna Maria Spagnolo, Michele Totaro, Angelo Baggiani, Gaetano Pierpaolo Privitera

**Affiliations:** 1 Department of Translational Research, N.T.M.S., University of Pisa, via San Zeno, 37/39-56127 Pisa, Italy; tuvobenedetta@hotmail.it (B.T.); michele.totaro.unipi@hotmail.com (M.T.); angelo.baggiani@med.unipi.it (A.B.); gaetano.privitera@med.unipi.it (G.P.P.); 2 Department of Health Sciences, University of Genoa, via Pastore, 1-16132 Genoa, Italy; cristinaml@unige.it (M.L.C.); am.spagnolo@unige.it (A.M.S.)

**Keywords:** healthcare-associated infections, hospital environmental cleaning and disinfection, ultraviolet C light-emitting device, high-touch surfaces

## Abstract

Implementation of environmental cleaning and disinfection has been shown to reduce the incidences of healthcare-associated infections. The effect of an enhanced strategy for terminal room disinfection, applying the pulsed xenon-based ultraviolet light no-touch disinfection systems (PX-UVC) after the current standard operating protocol (SOP) was evaluated. In a teaching hospital, the effectiveness in reducing the total bacterial count (TBC) and in eliminating high-concern microorganisms was assessed on five high-touch surfaces in different critical areas, immediately pre- and post-cleaning and disinfection procedures (345 sampling sites). PX-UVC showed only 18% (15/85) of positive samples after treatment compared to 63% (72/115) after SOP. The effectiveness of PX-UVC was also observed in the absence of manual cleaning and application of a chemical disinfectant. According to the hygienic standards proposed by the Italian Workers Compensation Authority, 9 of 80 (11%) surfaces in operating rooms showed TBC ≥15 CFU/24 cm^2^ after the SOP, while all samples were compliant applying the SOP plus PX-UVC disinfection. *Clostridium difficile* (CD) spores and *Klebsiella pneumoniae* (KPC) were isolated only after the SOP. The implementation of the standard cleaning and disinfection procedure with the integration of the PX-UVC treatment had effective results in both the reduction of hygiene failures and in control environmental contamination by high-concern microorganisms.

## 1. Introduction

The role of healthcare workers (HCW) in the transmission of pathogens from patient-to-patient is well documented; however, increasing evidence reports the contaminated environment as highly significant in pathogen transmission; in particular, high-touch surfaces are recognized as a possible reservoir of infectious agents and their contamination can pose a risk also for the spread of multi-resistant organisms [1,2,3,4]. High touch near-patient surfaces have actually higher bioburden and can contribute to secondary transmission by the direct contact with the patient or via the hands of HCW and visitors [5,6].

The relevance of the decontamination of the environment, such as patient-care rooms before admission of subsequent occupants, has therefore grown in recent years and high-touch sites are recommended to be cleaned and disinfected on a more frequent schedule than minimal touch surfaces [7].

Environmental cleaning and disinfection are important components of a comprehensive strategy in order to control healthcare-associated infections [8,9], especially in wards with immuno-compromised patients. However, studies evaluating the effectiveness of improved cleaning interventions have reported that approximately 5–30% of surfaces remain potentially contaminated, due to the inability of existing detergent formulations and disinfectants to disrupt biofilms [10,11]. Dry-surface biofilms on clinical surfaces were recently investigated and the survival of vegetative bacteria for long periods has been demonstrated [12,13].

There has been a lot of interest in the development of effective and more comprehensive environmental disinfection strategies and, in the last year, attention has been focused on improving “no touch” technologies, including the use of the mobile UV-light disinfection system, which has the advantages of not requiring changes in a room’s ventilation, not leave residue after treatment, and having a broad spectrum of action and rapid exposure times. The germicidal effects of UVC irradiation results in cellular damage by photohydration, photosplitting, photodimerization, and photocrosslinking, thereby inhibiting cellular replication. UVC can be generated from low-pressure mercury lamps that produce continuous UVC with a peak wavelength of 254 nm, and pulsed xenon lamps that emit pulsed light at high intensity, both in the spectrum of UVC (100–280 nm) and visible (380–700 nm) radiation, with a much broader microbicidal activity spectrum [14]. The UV-light disinfection system must operate in unoccupied rooms, after the patient discharge and in the absence of health personnel. Many devices have motion sensors that shut-off the device if any movement is detected inside the room being disinfected. Damage to materials in the room was not reported during the use of UV-light disinfection systems, although in the Pulsed-UVC device operator manual, high pressure acrylic material may show degradation for prolonged periods of exposure to light UV (e.g., daily or weekly), therefore it is advised to cover them during the treatment.

Implementation of this “no-touch” technology in various hospitals has documented a sustained reduction in surface microbial contamination, reduced cross contamination, and a reduced spread of multi-drug resistant bacterial infections. In the study of Liscynesky et al. [15], in rooms of patients with confirmed *C. difficile* infection (CDI), 32 out of 238 (13%) high-touch surfaces were positive after bleach disinfection and only 1 out of 238 (0.4%) was positive after UVC-treatment (the computer keyboard) at 254 nm emitted by 3 connected devices run for 45 min. Wong et al. reported the persistence environmental contamination by methicillin-resistant *Staphylococcus aureus* (MRSA), vancomycin-resistant Enterococcus (VRE) and *C. difficile*, respectively in 27%, 29,5%, and 22,7% of sites after the standard cleaning and disinfection protocol, whereas only in 3.3%, 4.9%, and 0% after UVC-disinfection (*p* < 0.05). The exposition time varied from 14 min at 46,000 µWs/cm^2^ to 57 min at 22,000 µWs/cm^2^ for the sporicidal cycle. The ability to disinfect high concentrations of organisms varies in the presence of proteins [16]. The same finding was reported by Ali et al., who observed lower and more variable log_10_ reductions in MRSA and *K. pneumoniae* after UVC disinfection at 254 nm when heavy soiling was present [17].

An increased reduction of 17% in MRSA, VRE, *Acinetobacter* spp., and carbapenem-resistant Enterobacteriaceae was reported by Hosein after 20 min pulsed xenon-based ultraviolet light disinfection (one device, two cycles) in addition to standard end-of-day manual cleaning [18].

Haddad et al. showed that combining standard between-case manual cleaning of surfaces, followed by a 2-min cycle of disinfection using a portable xenon pulsed ultraviolet light germicidal device, furtherly decreased the bacterial load by at least 70% [19]. The effectiveness of the pulsed xenon-based ultraviolet light systems in reducing aerobic bacteria, also in the absence of manual disinfection, was demonstrated by Jinadatha et al. [20].

Although some studies have reported doses of UVC that yield 3 log_10_ reductions of specific pathogens using low-pressure mercury lamps UVC devices, data regarding spectrophotometrically determined doses of 200–320 nm light emitted by pulsed xenon lamps are lacking [21].

Hospitals that use UV-light disinfection after cleaning and disinfection standard protocol have actually significantly mitigated infection risks associated with environmentally mediated transmission routes. In the BETR (Benefits of Enhanced Terminal Room Disinfection) study, the first randomized multicenter trial that compared the effectiveness of different disinfection strategies in rooms previously occupied by colonized/infected patients with the incidence of new colonization and infections in new hospitalized patients, demonstrated that the addition of UVC disinfection treatment to the standard protocol had a direct protective effect on the risk of acquiring *C. difficile* and vancomycin-resistant Enterococci [22,23].

The aim of this study was to evaluate the effectiveness on the field of an ultraviolet C (UVC) light-emitting device in reducing environmental bacterial burden and the presence of pathogens when compared to the current standard operating protocol (SOP).

## 2. Materials and Methods

A prospective open-labelled cross-over study was conducted in a 1158-bed teaching hospital in Italy with a follow up duration of four months, from September 2017 to December 2017. To evaluate the effectiveness of pulsed xenon-based ultraviolet light no-touch disinfection systems (PX-UVC) in reducing environmental contamination, sampling was performed in different critical areas: 5 patient rooms, 2 Intensive Care Units (ICU) isolation rooms, and 9 operating theatres (OT).

The inclusion criteria for the patient rooms and ICU isolation rooms were: A single occupancy room, occupied for a minimum of 48 h by patient colonized/infected by high concern microorganisms (reported as an alert by the microbiology laboratory). In the hospital, a systematic surveillance for multi-drug resistant organism colonization was performed through weekly rectal swabs and/or bronchial aspirate sampling.

### 2.1. PX-UVC Device

The PX-UVC device (Xenex Disinfection Services, San Antonio, TX USA) uses a xenon flash lamp to generate high-energy, broad-spectrum ultraviolet and visible light (UVC 100–280 nm, visible 380–700 nm), in microsecond bursts (pulses) at 67 Hz. No touch UV technology is dependent on the distance between the lamp and the surface being disinfected. The inverse square law states that the doubling of distance between the lamp and the surface being disinfected will quadruple the time required for disinfection. The PX-UVC device uses 5-min disinfection cycles and multiple positions with minimal distances from high-touch surfaces. The manufacturer recommends that high-touch surfaces are within two meters of the lamp in order to achieve optimal efficacy. For patient rooms, the device requires one 5-min disinfection cycle on each side the patient bed and one cycle in the private bathroom (if applicable). For operating theatres, the device requires one 10-min disinfection cycle on each side of the operating bed. Due to the high-intensity broad-spectrum UV light, the device is operated in unoccupied rooms. There are motion sensors that shut-off the device if any movement is detected inside the room being disinfected. The PX-UVC device, like most UVC lamps, causes chemical reactions that increase the concentration of ozone in the air. When the robot is operated in accordance with the procedures, the ozone produced is far below the Occupational Safety and Health Administration OSHA short-term exposure limits (0.1 ppm/8 h), however the manufacturer recommends using the robot in rooms with a system of ventilation, where possible. The robot allows access to the room (a green light turns on) after a delay that allows the ozone to dissipate. In our study, rooms were aerated after using the robot.

### 2.2. Study Protocol

The OTs were selected based on their different turnover time: two OTs scheduled for 10 surgeries/day (endocrine surgery) and four OTs scheduled for two major surgeries/day (implant of orthopedic prostheses and organ transplants).

In this hospital, the cleaning services were outsourced. According to the contract and the standard operating protocol (SOP), in terminal disinfection the housekeeping staff applied a chlorine-based detergent, Antisapril Detergent 10%, Angelini, followed by a chlorine-based disinfectant, Antisapril Disinfectant 10%, Angelini (active chlorine 2800 mg/L), on furniture surfaces and electromedical devices. In rooms at discharge of patient with *Clostridium difficile* CD infection, Antisapril Disinfectant was applied at 18%. In operation rooms, the same protocol was performed by in-house auxiliary nurses.

Following the alternative protocol, after SOP, the auxiliary nurses expose the pulsed xenon-based ultraviolet light device, for two 5 min cycles for each bedside in the patient rooms and in the intensive care unit, whereas 10 min cycles were adopted for each surgical table side in operating theaters. Auxiliary nurses were trained on the proper use of the Pulsed-UVC device.

Baseline microbiologic samples were collected after patient discharge or after surgical activity and immediately after sanitization. In each setting, five high-touch surfaces (for the operating room—surgical table, tray table, anaesthetic machine, monitor, infusion pump, scialitic lamp, electrosurgery; for the Intensive Care Units ICU—hydrotherapy tank, tray table, monitor, patient bed, infusion pump; for patient rooms—patient bed, tray table, medication cart, call button, push button) were sampled after healthcare activity (5 samples in dirty condition), after standard operating protocol SOP (5 samples in clean condition), and after Pulsed-UVC disinfection (5 samples in improved clean condition). In the high turnover Operating Theatre OTs, between one procedure and the following one we tested only the efficacy of the Pulsed-UVC disinfection, because internal hospital policy only provides for a terminal cleaning/disinfection. Considering that the Pulsed-UVC treatment in OT was carried out several times a day, as control in the study, one not treated OT was included for each treated one. On this, OT samples were taken before and after the SOP. This has allowed us to eliminate any overestimates of treatment efficiency, due to the cumulative effect of UVC radiation.

According to ISO 14698-1, 55-mm diameter Rodac plates containing plate count agar (PCA) with neutralizers (VWR International PBI, Radnor, Pennsylvania, PA) were used for the total viable count (TVC) enumeration and violet red bile dextrose agar (VRBD), (Oxoid, Basingstoke, UK) for Gram-negative bacteria qualitative evaluation. Contact plates were incubated aerobically at 37 °C for 48 h.

Suspect *Acinetobacter* spp. or *Klebsiella* spp. were subcultured on chromID™ mSuperCARBA (bioMérieux, Marcy l’Etoile, Capronne, France) and identified by the API/ID32 Strep Miniature System (bioMérieux, Marcy l’Etoile, Capronne, France).

In the OT and ICU, the total microbial load and the presence of pathogens were evaluated according to the hygienic standards proposed by the Italian Workers Compensation Authority [24], whereas in patient rooms the evaluation was conducted according to the standard proposed by Dancer et al. [25], (for the ICU: ≤50 CFU/24 cm^2^ and absence of pathogens, for the operating theaters: ≤15 CFU/24 cm^2^ and absence of pathogens; ≤125 CFU/24 cm^2^ for patient rooms).

Microbiologic sampling was performed using Rodac contact plates of 24 cm^2^ (Oxoid, Basingstoke, United Kingdom) that were firmly pressed for 5 seconds on each surface. The plates were then incubated at 37 °C for 48 h.

To detect the presence of *C. difficile* spores, the sponge contact method (Sponge-Sticks, 3M St. Paul, MN) was applied. The sponge heads were aseptically placed into sterile stomacher bags (VWR International, Milan Italy) containing 20 mL neutralising solution (0.1% sodium thiosulfate, 3% Tween 80, 0.3% Lecithin), prepared in phosphate-buffered saline solution pre-sterilized (PBS, Sigma-Aldrich). An area of 10 × 10 cm was delimited by a sterile plastic template and then swabbed with the moistened sponge. After sampling, each sponge was returned to the bag in which it was moistened. The total volume of homogenized solution was aliquoted (1.5 mL) into centrifuge sterile tubes, and centrifugated to 8000× *g* for 20 min at 25 °C. The pelleted cells were suspended in 1 mL of PBS, and re-centrifugate at 6000× *g* for 15 min. All pellets were subsequently suspended in 500 µL of PBS and aliquots of 250 µL plated onto Brazier’s agar plate (Oxoid, Basingstoke, Hampshire, United Kindom). The plates were incubated at 37 °C under anaerobic condition for 48 h. Presumptive *C. difficile* isolates were determinate by colony morphology (examination of plates for flat, circular colonies yellow/grey in colour with filamentous edges) and confirmed to be *C. difficile* using *C. difficile* selective latex agglutination assays (Oxoid, Basingstoke, United Kingdom).

### 2.3. Statistical Analysis

To compare the number of hygiene failures and the total number of positive samples obtained after each cleaning and disinfection procedure we applied the Wilcoxon matched-pairs signed rank test to analyse the results obtained in patient rooms and ICUs, while for OTs low turnover and OTs high turnover the analysis was conducted with the Mann–Whitney test. Statistical significance was inferred from *p* < 0.05. Statistical analysis was performed using Prism 8 (GraphPad Software, San Diego, CA, USA).

## 3. Results

We sampled a total of 345 high-touch surfaces—135 after healthcare activity, 125 after SOP, 85 after application of SOP and Pulsed-UVC treatment. 20 samples were collected after Pulsed-UVC disinfection applied without perform SOP.

A total of 2339 colonies were isolated from environmental surfaces. All but 39 were consistent with skin commensal (106 were *Staphylococcus* spp.) and of these, 6 colonies of mold were grown, 29 Gram negative bacteria (three *Enterobacter cloacae*, one *Vibrio alginoliticus*, 10 *Cryseobacterium menigosepticum*, seven *Edwarsiella hoshinae*, two *Methylobacterium mesofilicum*, four KPC-*K. pneumoniae,* two Extended Spectrum *β* Lactamase-producing *Klebsiella pneumoniae* (ESBL-*K. pneumoniae))* and four bacillus identified as *C. difficile*. Before cleaning and disinfection, the average of CFUs was 6 ± 10 standard deviation (SD) CFU/24 cm^2^ in OTs with low turnover, 7 ± 12 SD CFU/24 cm^2^ in OTs with high turnover, 25 ± 19 SD CFU/24 cm^2^ in the ICUs, and 58 ± 54 SD CFU/24 cm^2^ in patient rooms at discharge.After SOP, the average of CFUs increased to 11 ± 18 SD CFU/24 cm^2^ in OTs with low turnover (+83%), while it decreased to 1 ± 1 SD CFU/24 cm^2^ (−7%) in OTs with high turnover, and in ICUs and patient rooms, respectively, to 2 ± 4 SD CFU/24 cm^2^ (−92%) and 8 ± 13 SD CFU/24 cm^2^ (−86%).

After Pulsed-UVC disinfection, approximately all the average of CFUs were 0 CFU/24 cm^2^: 0 ± 1 SD CFU/24 cm^2^ in ICUs (−100%), 1 ± 1 SD CFU/24 cm^2^ in patient rooms (−98%), 0 ± 1 SD CFU/24 cm^2^ in OTs with high turnover (−100%), and 0 ± 0 SD CFU/24 cm^2^ in OTs with low turnover (−100%).

As concerned, the increased reduction obtained after the Pulsed-UVC treatment was 12% in patient rooms, 8% in ICUs, 93% in OTs with low turnover, and 183% in OTs with high turnover.

The median, lower, and higher values of the bacterial load and the interquartile range obtained in each hospital setting are reported in Table 1.

After the application of SOP, 11% (9/80) surfaces in OTs with low turnover showed TBC ≥ 15 CFU/24 cm^2^ (hygiene failures) (4 infusion pumps, 2 scialitic lamps, 2 anaesthetic machines, 1 surgical table). The CFU average did not undergo significant variation after the application of SOP, rather we underlined an increase on the CFU amount. Probably, when the housekeeping combined detergent/hypochlorite treatment failed to eliminate the microbial contamination from the surface and the cleaning cloth was then used to wipe another surface, the bacterial was transferred to other surfaces and to the hands of the auxiliary nurses handling the cloth.

One hundred percent (10/10) of surfaces in OTs with high turnover were compliant after SOP and after Pulsed-UVC treatment without application of SOP (20/20) (*p* < 0.18; *n* = 20).

In the ICUs, 100% (10/10) of samples were found to be compliant already after application of SOP as well as after the Pulsed-UVC treatment (*p* < 0.16; *n* = 20) as for surfaces in patient rooms, 100% (50/50) compliant for TBC level (less than 125 CFU/24 cm^2^) after SOP (*p* < 0.0001; *n* = 50).

Before the surgical activity on OTs with high turnover, between one surgery and another, the SOP was not applied and 7 surfaces were non-compliant with the standard (1 tray table, 1 anaesthetic machine, 2 scialitic lamps, 2 electrosurgeries). The total number of non-compliant samples after application of the SOP were 9/115 (8%) against 0/85 (0%) after Pulsed-UVC treatment (*p* < 0.05).

**Figure 1:**
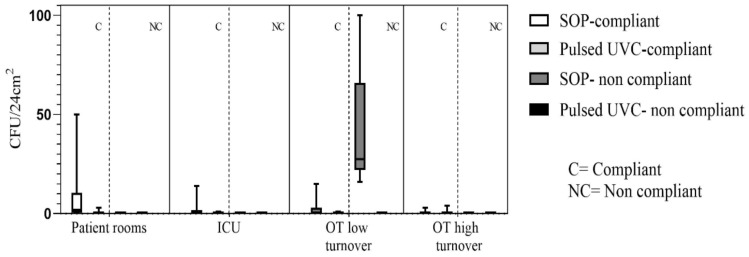
Median, lower, and higher values of the bacterial load detected in each hospital setting, distinguishing compliant on non-compliant values. Note: ICU—Intensive Care Unit; OT—Operative Theatre; C&D—Cleaning and Disinfection; SOP—Standard Operative Procedure; IQR—Interquartile Range.

The total number of positive samples, after the SOP, were found to be 72/115 (63%), whereas 15/85 (18%) after treatment with Pulsed-UVC (Figure 1) (*p* < 0.05).

### High Concern Microorganisms

During the studied period, in a room previously occupied by a patient in contact precautions due to gastrointestinal colonization by KPC-producing *K. pneumoniae*, we detected three surface sample positives for KPC-*K. pneumoniae* on the tray table, the bedside table and the nurse call bell respectively, after discharge of patient; the bedside table remained positive after application of SOP.

In a room where a patient with ESBL-*K. pneumoniae* gastrointestinal colonization was hospitalized for a week, after patient discharge, we detected ESBL-*K. pneumoniae* on a tray table, but not after SOP.

After discharge, *C. difficile* spores were detected on 4 surfaces out of 5 in a room where the patient was admitted for 3 days: Bed patient, tray table, call button and push button. After the application of SOP, the bed patient surface remained positive for *C. difficile* spores, that was no longer found after the application of the Pulsed-UVC treatment.

In conclusion, in the post-application of SOP, 96% (24/25) of surface samples were compliant with the absence of high concern microorganisms and 100% (25/25) after Pulsed-UVC treatment.

## 4. Discussion

The manual terminal cleaning of clinical areas is aimed to reduce the burden of microbial contamination, but often is not able to completely eliminate it [26,27] and furthermore there is growing evidence that the contaminated environment is highly significant in pathogen transmission leading to healthcare-associated infections (HCIs) [28,29]. Decontamination of the environment such as patient-care rooms before admission of subsequent occupants has therefore become of more importance in recent years and the assurance of sufficient decontamination more vital [30]; its importance was noted as pivotal in blocking norovirus and *C. difficile* transmission [31].

The role of housekeepers in a hospital is fundamental because they influence on the effectiveness of cleaning disinfection practice. Their high turnover, incorrect disinfectant contact times, and over-dilution of disinfectant solutions are negative factors for successful cleaning [32].

Numerous studies have demonstrated that current strategies for terminal room disinfection are inadequate and 50% or more hospital surfaces may go untouched and uncleaned following terminal room disinfection [3].

Cleaning is a complex, multifaceted process, plagued with random variation and the potential for introducing pathogens if cleaning cloths and solutions become contaminated and not correctly used.

Extensive outsourcing of hospital cleaning services to private sector contractors that employ staff with precarious working conditions and hence low motivation, achieving lower levels of cleanliness than the in-house staff, is frequent in Italy as well as in other countries. In-house staff have more perception on the importance of the cleaning in reducing the environmental microbial contamination. Toffolutti et al. found that outsourcing cleaning services was associated with greater incidence of MRSA and worse patient perceptions of cleanliness [33].

Pre-impregnated disinfectant cloths are used in an attempt to increase the efficacy of cleaning, because the sanitization process is faster and easier with a consequent increase in cleaning staff compliance. The effectiveness depends on the active ingredients they contain and its quantity, as well as the method of use [34,35]. The microfibre cloths remove more bacteria than cotton and synthetic fibre cloths [36]. Improper use of wipes could spread potential pathogens across surfaces if a “1 wipe, 1 application” per surface policy is not adopted [37].

In the last few years, no-touch systems for environmental decontamination are increasingly being considered, such as the UVC no-touch technology that can be done routinely and rapidly in different hospital settings after patient discharge or transfer.

In our study, we found Pulsed-UVC disinfection effective in reducing microbial contamination, showing only 18% (15/85) of positive samples after treatment compared to 63% (72/115) after SOP, and 12% increased reduction of positive samples in patient rooms, 8% in ICUs, 93% in OTs with low turnover, and 183% in OTs with high turnover. The treatment effectiveness was observed also in absence of any manual cleaning and application of a chemical disinfectant. In OT with high turnover, between one surgical operation and another, the standard protocol was not applied, and although the average bacterial load detected before the cleaning and disinfection procedures was low (7 ± 12 SD CFU/24cm^2^), 13 sampling sites out of 20 showed bacterial load, three sites over 15 CFU/24 cm^2^. Pulsed-UV disinfection reduced aerobic bacteria in the absence of manual cleaning and disinfection. The same results were obtained in a study conducted by Jinadatha et al. where Pulsed-UV disinfection effectively reduced MRSA colony counts in the absence of manual disinfection and the authors suggested the use of Pulsed-UV disinfection as an adjunct to existing terminal cleaning protocols since it offers a safety net when the primary approaches fail [20].

Our study is one of the few in which the effectiveness of Pulsed-UVC on surfaces of hospital settings where manual cleaning was not performed was evaluated. Several studies [16,17] have demonstrated that the effectiveness of continuous UVC produced by low-pressure mercury lamps systems is diminished with increasing concentrations of organic or protein matter. The effectiveness of high-energy, broad-spectrum light produced by the Pulsed-UV system has not been shown to be affected by the lack of manual surface cleaning. Our results confirmed that this no-touch technology does not replace the traditional manual terminal cleaning and disinfection protocol, but it can improve it when a surface was missed by the housekeeping staff. Pulsed-UVC disinfection can be an excellent adjunct to the standard cleaning protocol, but it is important that infection preventionists maximize its usage to achieve the most efficiency, taking into account the facility’s patient flow and operational needs, to obtain a return on the investment cost.

Our study had several limitations. This is an experimental study and the hospital where it was conducted, despite being available for experimentation, had to reconcile the delays caused by the application of the protocol to the healthcare activity. In particular, the number of surfaces sampled after Pulsed-UV exposure was not high due to the difficulty of applying the treatment in operating theaters, where the scheduling of surgical activity cannot be delayed. Moreover, only isolation single rooms in ICU were included in the study, since the treatment was not applicable in multi-bed rooms.

In this regard, our proposal is to use this approach of implementing environmental disinfection in hospital rooms or in ICUs at patient discharge. In operating theaters, the possibility of exposure times reduced to few minutes should be considered, as already demonstrated by Haddad et al. [19].

## 5. Conclusions

No-touch surface decontamination technologies that use ultraviolet light may be effective in enhancing the results of the effort spent to reduce the microbial burden and potentially achieving lower Healthcare-associated Infections HAIs rates, as aimed for in infection control strategies.

Hence, these data are important for hospitals that plan to adopt this technology as adjunct to routine manual disinfection; providing the goal is to eliminate surface bioburden and as a consequence, HAIs, hospitals will need to continue to improve in both hand hygiene and environmental disinfection.

In conclusion, Pulsed-UV technology was effective at reducing overall bacterial counts and significantly more successful than manual disinfection alone on hospital surfaces. Further evaluation focusing on clinically meaningful reduction in HAIs is of paramount importance in justifying the cost and effort in implementing this promising technology in the battle against pernicious hospital infections. Our results underline important critical issues in standard terminal cleaning (combined manual cleaning and chemical disinfection) on high touch surfaces, to adequately remove microbial contamination from the environment.

We have demonstrated that the Pulsed-UVC device, associated with SOP, significantly reduced microorganisms from common high-touch surfaces.

## Figures and Tables

**Table 1:** Median, lower, and higher values of the bacterial load detected in each hospital setting.

Setting	Timing of Sampling	*n* (Samles)	Median	Lower	Higher	IQR
Patient rooms	Before C&D	25	43	0	180	93
	After SOP	25	2	0	50	7
	After SOP + Pulsed-UVC	25	0	0	3	1
ICU	Before C&D	10	23	1	50	45
	After SOP	10	1	0	14	2
	After SOP + Pulsed-UVC	10	0	0	1	0
OT low turnover	Before C&D	60	1	0	100	4
	After SOP	80	1	0	100	6
	After SOP + Pulsed-UVC	30	0	0	1	0
OT high turnover	Before C&D	40	7	0	38	25
	After SOP	10	0	0	3	1
	After Pulsed-UVC	20	0	0	4	0

Note: ICU—Intensive Care Unit; OT—Operative Theatre; C&D—Cleaning and Disinfection; SOP—Standard Operative Procedure; IQR—Interquartile Range.
